# Impact of aversive affect on neural mechanisms of categorization decisions

**DOI:** 10.1002/brb3.3312

**Published:** 2023-11-15

**Authors:** Daniel J. Levitas, Kess L. Folco, Thomas W. James

**Affiliations:** ^1^ Department of Psychological and Brain Sciences Indiana University Bloomington Indiana USA

**Keywords:** decision‐making, distractor effect, drift‐diffusion model, evidence accumulation

## Abstract

**Introduction:**

Many theories contend that evidence accumulation is a critical component of decision‐making. Cognitive accumulation models typically interpret two main parameters: a drift rate and decision threshold. The former is the rate of accumulation, based on the quality of evidence, and the latter is the amount of evidence required for a decision. Some studies have found neural signals that mimic evidence accumulators and can be described by the two parameters. However, few studies have related these neural parameters to experimental manipulations of sensory data or memory representations. Here, we investigated the influence of affective salience on neural accumulation parameters. High affective salience has been repeatedly shown to influence decision‐making, yet its effect on neural evidence accumulation has been unexamined.

**Methods:**

The current study used a two‐choice object categorization task of body images (feet or hands). Half the images in each category were high in affective salience because they contained highly aversive features (gore and mutilation). To study such quick categorization decisions with a relatively slow technique like functional magnetic resonance imaging, we used a gradual reveal paradigm to lengthen cognitive processing time through the gradual “unmasking” of stimuli.

**Results:**

Because the aversive features were task‐irrelevant, high affective salience produced a distractor effect, slowing decision time. In visual accumulation regions of interest, high affective salience produced a longer time to peak activation. Unexpectedly, the later peak appeared to be the product of changes to both drift rate and decision threshold. The drift rate for high affective salience was shallower, and the decision threshold was greater. To our knowledge, this is the first demonstration of an experimental manipulation of sensory data or memory representations that changed the neural decision threshold.

**Conclusion:**

These findings advance our knowledge of the neural mechanisms underlying affective responses in general and the influence of high affective salience on object representations and categorization decisions.

## Graphical abstract

We investigated the influence of affective salience on neural evidence accumulation parameters. Time series in visual accumulator regions of interest (ROIs) showed a later peak with high salience, the result of a shallower drift rate, and higher decision threshold. Time series in the amygdala were only sensitive to high salience in the post‐decision period.

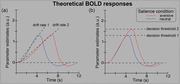

## 1 INTRODUCTION

Decision‐making plays a critical role in producing behavioral responses to our surroundings. Many theories contend that a critical component of decision‐making is the accumulation of relevant information or evidence, a cognitive process that has been extensively examined using mathematical modeling of behavioral measures (Audley & Pike, 1965; Link & Heath, 1975; Peters & D'Esposito, 2020; Ratcliff & Rouder, 1998; Ratcliff et al., 2016; Usher & McClelland, 2001). For decades, general classes of evidence accumulation models (commonly expressed by drift‐diffusion models) have been used to formally assess the cognitive mechanisms of decision‐making under different experimental conditions by assigning quantitative parameters to its multiple aspects (Laming, 1968; Ratcliff, 1978; Smith & Ratcliff, 2004; Stone, 1960). More recently, researchers have begun searching for neural markers of accumulation of evidence that may signify mechanisms involved in decision‐making (Carlson et al., 2006; Gold & Shadlen, 2007; Hanes & Schall, 1996; Heekeren et al., 2004; James et al., 2000; Ploran et al., 2007; Schall, 2019; Shadlen & Newsome, 2001).

Although many variations of evidence accumulation models exist, the vast majority emphasize two key parameters: a decision threshold and a drift rate. The decision threshold represents the amount of evidence necessary to make a decision given the context of the task, namely, the emphasis on accuracy or speed (for review, see Gold & Shadlen, 2007; Hanes & Schall, 1996; Heekeren et al., 2004; James et al., 2000; Ploran et al., 2007; Schall, 2019; Shadlen & Newsome, 2001). In other words, the decision threshold represents the stopping rule. Although this threshold is relatively stable in decision paradigms, it has been shown to vary across situations, primarily when speed is prioritized over accuracy (Bogacz et al., 2006; Ratcliff et al., 2016). The drift rate is the rate at which evidence is accumulated and is reflective of the quality of evidence, either contained in the sensory data or the memory representation (Ratcliff & McKoon, 2008). Specifically, the slope of the drift diminishes when the quality of evidence is poor.

Although much less extensive than the behavioral literature, a limited number of neural investigations point to the accumulation of evidence being not just a description of behavior, but possibly an underlying neural mechanism of decision‐making. Electrophysiological measurements in animal models have been most successful at resolving how pre‐decision activity contributes to the subsequent behavioral response (Hunt et al., 2015; Kim & Shadlen, 1999; Shadlen & Newsome, 2001; Thompson et al., 1996). Using non‐invasive functional magnetic resonance imaging (MRI) methods on humans, however, poses a significant challenge because of the discrepancy between the slow temporal resolution of blood oxygenation‐level dependent (BOLD) measurements and the rapid dynamics of the types of decisions commonly used in human cognitive studies, such as object recognition/decision (Logothetis & Sheinberg, 1996; Potter, 1976; Thorpe et al., 1996). Fortunately, previous functional MRI (fMRI) studies have developed gradual reveal paradigms that “unmask” visual stimuli over a long time period and act as an artificial means of lengthening the processing time required for recognition/decision (Carlson et al., 2006; Eger et al., 2007; James et al., 2000; Kleinschmidt et al., 2002; Ploran et al., 2007; Wheeler et al., 2008). Such paradigms provide researchers with some ability to separately analyze different time periods of a decision trial and, most importantly, dissociate activation that occurred before or after a decision.

Previous use of the gradual reveal paradigm combined with fMRI has elucidated multiple temporal activation profiles—the temporal pattern of activation across a decision trial—in different brain regions and correlated them to perceptual and cognitive processes. The most thorough of these was a 2007 article by Ploran et al. (2007) that used a hierarchical clustering algorithm to classify temporal profiles (and hence brain regions) based on their positive, negative, bimodal, or late positive signatures. Positive profiles resembled evidence accumulators, which had been previously described by James and colleagues (James et al., 2000; Ploran et al., 2007), that is, a gradual increase in activation with a peak at the decision time, and with shallower slopes for longer decisions; negative profiles resembled inverted accumulators; bimodal profiles had an initial short period of increased activation followed by a short period of decreased activation, both before the decision time; and late positive profiles seemed uninfluenced by decision time and showed baseline activation until the end of the trial at which time they increased. A second analysis subdivided the large set of positive profiles into three distinct classes: a sensory profile that essentially increased to asymptote while the stimulus was presented, an evidence accumulator profile that resembled the overall positive profile, and a moment‐of‐recognition/decision profile that largely remained at baseline, except for a spike of activation right at the decision time (Carlson et al., 2006; Eger et al., 2007; James et al., 2000; Kleinschmidt et al., 2002; Ploran et al., 2007; Wheeler et al., 2008). Although the functional significance of the evidence accumulator profile is supported by a wealth of cognitive modeling research, the other profiles—sensory, moment‐of‐decision (MoD), negatives, and so on—suggest that the gradual revealing paradigm has untapped potential for understanding the neural mechanisms underlying decision‐making and other cognitive processes.

Additionally, regions that exhibited the evidence accumulation profile were able to be analyzed with the key drift‐diffusion parameters: drift rate (slope) and decision threshold (peak magnitude). For example, James et al. (2000) used a gradual reveal paradigm combined with a priming task. As expected, recognition/decision times were faster for primed objects. Neurally, faster decisions were related to a quicker rise to peak activation in accumulator regions of interest (ROIs). This was due to the combination of a steeper drift rate for primed objects but the same decision threshold for both primed and non‐primed objects. Relating these results to cognitive models suggests that priming improved the quality of the evidence available in memory and did not change the confidence necessary for recognition (i.e., the stopping rule). Despite the important links between neural and cognitive function that the study provided, it seems to represent the only experimentally controlled manipulation of decision time using a gradual reveal paradigm in the literature.

Here, we wanted to follow up on the study by James et al., but influence decision times by manipulating a factor other than recency (priming). It is known that the facilitatory influence of priming is based on changes to memory representations (Ratcliff & McKoon, 1988; Schacter & Buckner, 1998). The drift rate in drift‐diffusion models can be influenced by the quality of evidence extracted from a memory representation but can also be influenced by the quality of evidence extracted from the sensory data. The study by James et al. held the sensory data constant across conditions and only varied the memory representation. We wanted to test what influence changing the sensory data would have on neural accumulation, settling on manipulation of affective salience.

Affective salience refers to sensory information or evidence that stands out due to its emotionally arousing qualities and has been found to affect many aspects of perceptual experiences (Lim et al., 2009; Markovic et al., 2014; Todd et al., 2012). It has been proposed that the evolutionary benefit of quickly detecting and reacting to such salient information (e.g., predator detection) means that such processing occurs rapidly (Globisch et al., 1999). The importance of affective salience is so pronounced that a functional salience network (known anatomically as the midcingulo‐insular network) has been proposed: a collection of interlinked cortical and subcortical regions sensitive to salient information and responsible for the communication and allocation of resources across different brain networks (Goulden et al., 2014; Menon & Uddin, 2010; Seeley et al., 2007; Sridharan et al., 2008). Thus, behavioral and neuroimaging findings show that the brain is finely tuned to detect high affective salience in the environment and that the processing of affectively‐laden information interacts with various perceptual and cognitive processes.

Specifically, a significant body of research has demonstrated that decisions made about objects with high affective salience are different from those with low affective salience, suggesting that the affective characteristics of stimuli may alter evidence accumulation (Gray et al., 2002; Hodsoll et al., 2011; Phelps et al., 2006; Schupp et al., 2003; Thielscher & Pessoa, 2007; Vuilleumier & Driver, 2007). Even more specifically, affective characteristics that are unrelated to the task at hand can result in a distractor effect, a phenomenon by which attention is briefly orientated to task‐irrelevant information as opposed to task‐relevant information (Hartikainen et al., 2000; Tipples & Sharma, 2000). Behaviorally, this results in decreased accuracy and longer reaction times. From an evidence accumulation perspective, a distractor effect suggests a reduction in the quality of information that can be extracted from the sensory data, leading to a shallower drift rate. Alternatively, affectively laden stimuli may lead to a more conservative stopping rule by raising the decision threshold.

**FIGURE 1:**
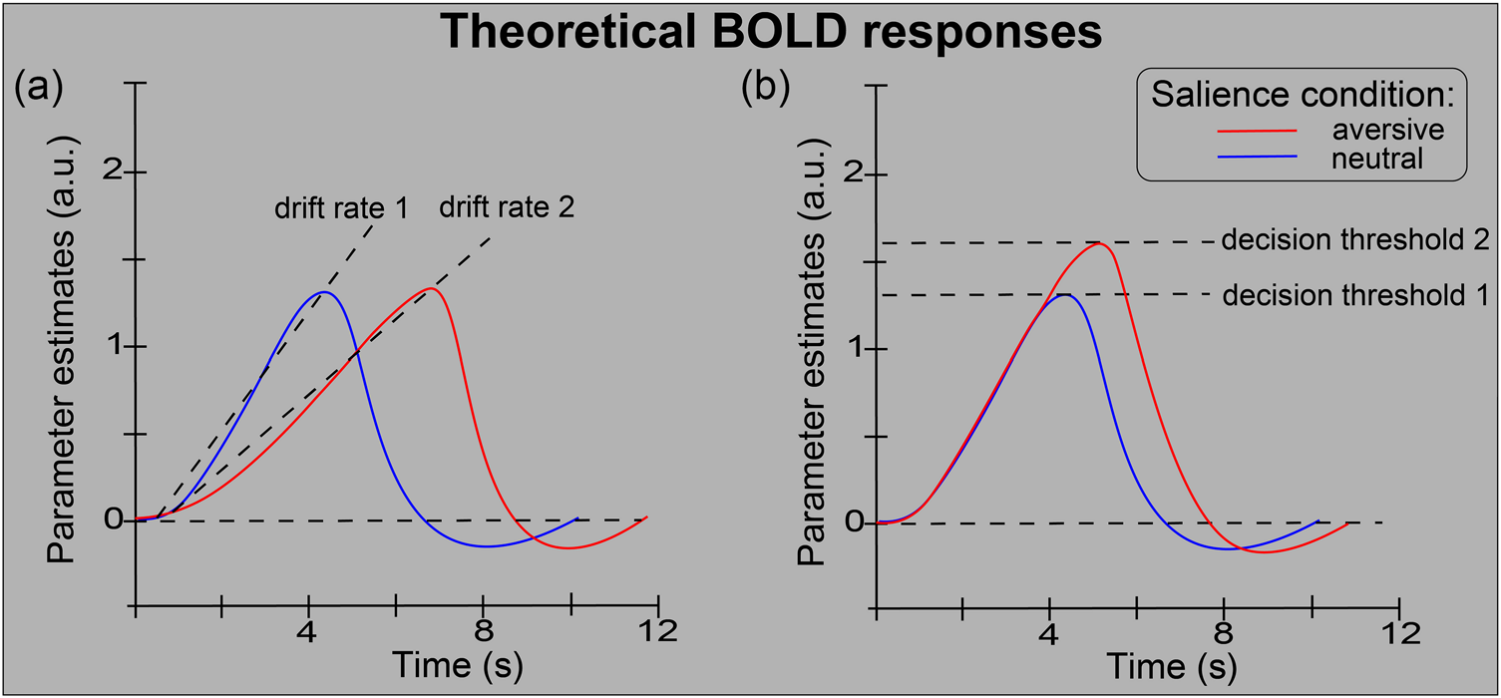
Theoretical predictions of the effect of high affective salience on evidence accumulation temporal profiles. (a) Example profiles if affective salience influences the drift rate (slope) but not peak magnitude (i.e., decision threshold) parameter of the accumulation process. (b) Example profiles if affective salience influences the peak magnitude (i.e., decision threshold) but not the drift rate (slope) parameter of the accumulation process. a.u., arbitrary units.

The primary aim of this study was to assess the influence of high affective salience on object categorization decisions by examining its influence on neural evidence accumulation. Theoretical predictions of the effect of affective salience on accumulation profiles are shown in Figure 1. Figure 1a reflects the situation where high affective salience (through a distractor effect) decreases the drift rate (slope) but has no influence on decision threshold (peak magnitude). With no difference in the magnitude of peak activation between affective salience conditions, a shallower slope will mean that the peak will occur later in the trial for high affective salience. The later peak should coincide with longer decision times, as previously demonstrated in the neuroimaging literature (James et al., 2000; Ploran et al., 2007) and behavioral literature (Ratcliff, 1978; Smith & Ratcliff, 2004). An alternative hypothesis is shown in Figure 1b. It is well documented that high affective salience increases activation in many cortical regions, including some in visual cortex (Barrett & Bar, 2009; Lang et al., 1998; Schupp et al., 2003). A hypothesis consistent with these findings would be an increase in the magnitude of peak activation for high affective salience, as shown in Figure 1b. This could occur in the absence of any change in the drift rate and would reflect a change in the decision threshold, with high affective salience producing a more conservative stopping rule.

## 2 MATERIALS AND METHODS

### 2.1 Stimuli

Stimuli consisted of 100 images (and an additional six for practice) with 50 images of feet and 50 images of hands. Half of the appendages contained highly affective salient features, such as lacerations and mutilations. All images were resized to 600 × 600 pixels, with gray‐scaled backgrounds in order to keep the emphasis on the appendage. The viewing distance from the display mirror to eyelid was 11.5 cm, and the distance from the screen to the mirror was 79 cm, giving a total viewing distance of 90.5 cm. When projected in this manner, the size of the entire 600 × 600 pixels stimulus image subtended ∼13° of visual angle. The experiment was displayed on a Mitsubishi LCD projector (model XL30U), with a resolution of 1920 × 1200 pixels.

### 2.2 Participants

Thirty‐seven adults aged 18–58 (13 males; average age = 23.24 years, SD = 6.90) with normal or corrected‐to‐normal vision were recruited to participate in the study, which was approved by the Institutional Review Board of Indiana University. One participant was removed from analysis due to being notably older (age = 58) than the rest of the study cohort. Thus, the remaining cohort was 36 participants aged 18–31 (12 males; average age = 22.3 years, SD = 3.68 years). Participants were compensated $35.

### 2.3 Experimental paradigm

**FIGURE 2:**
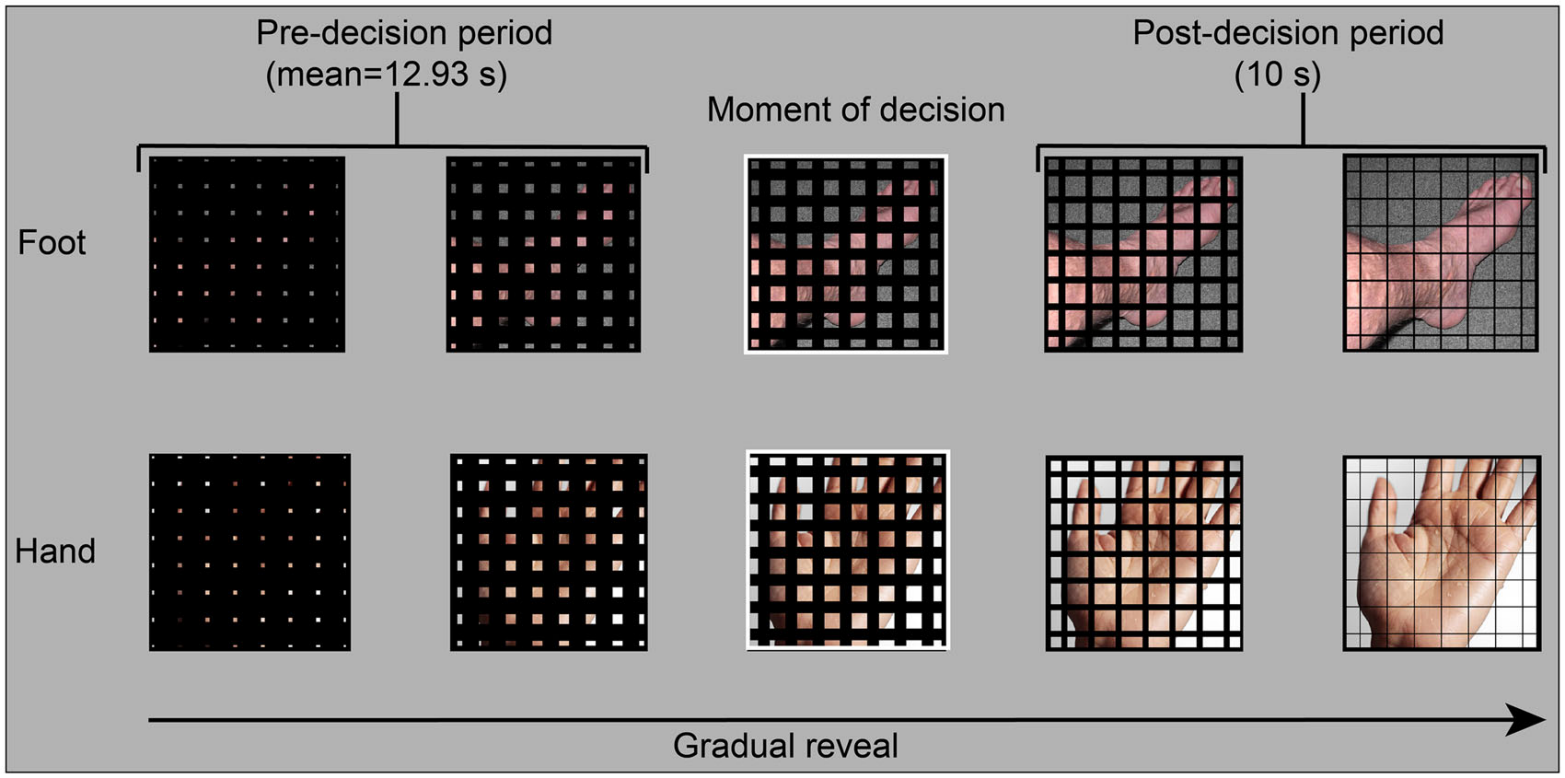
Example trial sequence of experimental paradigm. Participants were instructed not to respond until they felt confident they could categorize the image. Once a response was made (moment‐of‐decision), the gradual reveal continued for an additional 10 s (post‐decision period). If the reveal completed before the post‐decision 10‐s mark, the fully revealed image remained on screen until the 10‐s duration was reached.

Participants completed a gradual reveal perceptual categorization task (Figure 2). At each trial onset, an image was completely occluded by 14 overlayed black bars: seven vertical and seven horizontal. Over the course of 25 s, the width of the bars gradually eroded, revealing the image underneath. During this period, participants were instructed to categorize the image as either a hand or a foot. Decision responses for hand images were made with a button press of the right hand index finger and a button press of the left hand index finger for a foot image. Participants were instructed to only make a decision response once they felt “confident” they could categorize the stimulus in which the accuracy was of greater importance than speed. Once participants had responded, the gradual reveal continued for an additional 10 s. The image remained for the entire 10 s, even if the gradual reveal had reached completion. This was followed by a jittered 6‐, 8‐, or 10‐s intertrial interval. Trial order was randomized for each subject.

The experiment was administered using PsychoPy version 1.85.6 (Peirce, 2008). Participants completed six to nine runs of the task, each run lasting 6 min. Trials were self‐paced, such that trial lengths varied based on the amount of time it took participants to categorize each image. As such, not all participants completed the entire 100 trials within the allotted scan time. On average, between 92 and 93 trials were completed (min = 65, max = 100). Prior to the functional acquisition and during the acquisition of the T1‐weighted anatomical volume, participants completed a brief 2‐ to 3‐min practice session comprising six trials similar to those described above to familiarize them with the task. The stimulus images used for the practice trials were not used again for the trials during the functional acquisition.

### 2.4 Image acquisitions

Imaging data were collected on a Siemens Tesla 3T Prisma, whole‐body MRI system using a 64‐channel head coil. A T1‐weighted anatomical volume was acquired (TR = 2400 ms, TE = 2.68 ms, flip angle = 8°, inversion time = 1060 ms, and 0.8 × 0.8 × 0.8 mm isometric voxel size). This was followed by a spin‐echo field mapping sequence to reduce magnetic field inhomogeneities (Jezzard & Clare, 1999), comprising two brief acquisitions with opposite phase‐encoding directions. Functional data consisted of using T2*‐weighted BOLD interleaved echo‐planar imaging (EPI) sequences (TR = 420 ms, TE = 30 ms, multiband factor = 8, 48 slices, and 3 mm isotropic voxels). Each functional acquisition was preceded by a single‐band reference (SBRef) to be used as a reference template for motion correction. Due to the emphasis on temporal dynamics in the proposed analysis, the spatial resolution of the functional acquisitions was reduced to maximize the temporal resolution. A T2‐weighted anatomical volume was also acquired (TR = 3200 ms, TE = 564 ms, flip angle = 120°).

### 2.5 Data analysis

Imaging data were preprocessed using fMRIPrep 20.2.0 LTS (Esteban et al., 2019). The anatomical and functional preprocessing sections are described verbatim from the fMRIPrep reports, recommended by the developers to ensure transparency and future reproducibility.

#### 2.5.1 Anatomical

The T1‐weighted (T1w) image was corrected for intensity non‐uniformity with *N4BiasFieldCorrection* (ANTs 2.3.3) and used as T1w‐reference throughout the workflow (Tustison et al., 2010). The T1w‐reference was then skull‐stripped with a *Nipype* implementation of the antsBrainExtraction.sh workflow (from ANTs), using OASIS30ANTs as target template. Brain tissue segmentation of cerebrospinal fluid (CSF), white matter (WM), and gray matter was performed on the brain‐extracted T1w fast (FSL 5.0.9). Brain surfaces were reconstructed using *recon‐all* (FreeSurfer), and the brain mask estimated previously was refined with a custom variation of the method to reconcile ANTs‐derived and FreeSurfer‐derived segmentations of the cortical gray matter of Mindboggle (Klein et al., 2017). Volume‐based spatial normalization to the MNI152NLin6Asym standard space (*FSL's MNI ICBM 152 non‐linear 6th Generation Asymmetric Average Brain Stereotaxic Registration Model*) was performed through nonlinear registration with *antsRegistration* (ANTs 2.3.3), using brain‐extracted versions of both T1w reference and the T1w template.

#### 2.5.2 Functional

For each of the eight to nine BOLD runs found per subject (across all tasks and sessions), the following preprocessing was performed. First, a reference volume and its skull‐stripped version were generated by aligning and averaging 1 SBRef. A deformation field to correct for susceptibility distortions was estimated based on fMRIPrep's *fieldmap‐less* approach. The deformation field is that resulting from co‐registering the BOLD reference to the same‐subject T1w‐reference with its intensity inverted. Registration was performed with *antsRegistration* (ANTs 2.3.3) and modulated with an average fieldmap template, and the process was regularized by constraining deformation to be nonzero only along the phase‐encoding direction. Based on the estimated susceptibility distortion, a corrected EPI reference was calculated for a more accurate co‐registration with the anatomical reference. The BOLD reference was then co‐registered with the T1w reference using *bbregister* (FreeSurfer) which implements boundary‐based registration (Greve & Fischl, 2009). Co‐registration was configured with six degrees of freedom. Head‐motion parameters with respect to the BOLD reference (transformation matrices, and six corresponding rotation and translation parameters) were estimated before any spatiotemporal filtering using *mcflirt* (FSL 5.0.9). First, a reference volume and its skull‐stripped version were generated using a custom methodology of *fMRIPrep*. The BOLD time series were resampled onto their original, native space by applying a single, composite transform to correct for head‐motion and susceptibility distortions. These resampled BOLD time series are referred to as *preprocessed BOLD in original space*, or just *preprocessed BOLD*. The BOLD time series were resampled into standard space, generating a *preprocessed BOLD run in MNI152NLin6Asym space*. First, a reference volume and its skull‐stripped version were generated using a custom methodology of *fMRIPrep*. Automatic removal of motion artifacts using independent component analysis (ICA‐AROMA) was performed on the *preprocessed BOLD on MNI space* time series after the removal of non‐steady state volumes and spatial smoothing with an isotropic, Gaussian kernel of 6 mm FWHM (full‐width half‐maximum). Corresponding “non‐aggresively” denoised runs were produced after such smoothing. Three global signal time series were calculated based on the *preprocessed BOLD*, extracted within the CSF, the WM, and the whole‐brain masks. Confound time series derived from head motion estimates and global signals were expanded with the inclusion of temporal derivatives and quadratic terms for each (Satterthwaite et al., 2013). All resamplings can be performed with *a single interpolation step* by composing all the pertinent transformations (i.e., head‐motion transform matrices, susceptibility distortion correction when available, and co‐registrations to anatomical and output spaces). Gridded (volumetric) resamplings were performed using *antsApplyTransforms* (ANTs), configured with Lanczos interpolation to minimize the smoothing effects of other kernels (Lanczos, 1964). The ICA‐AROMA denoised data were used for all subsequent analyses (Pruim et al., 2015).

#### 2.5.3 fMRI analysis

**FIGURE 3:**
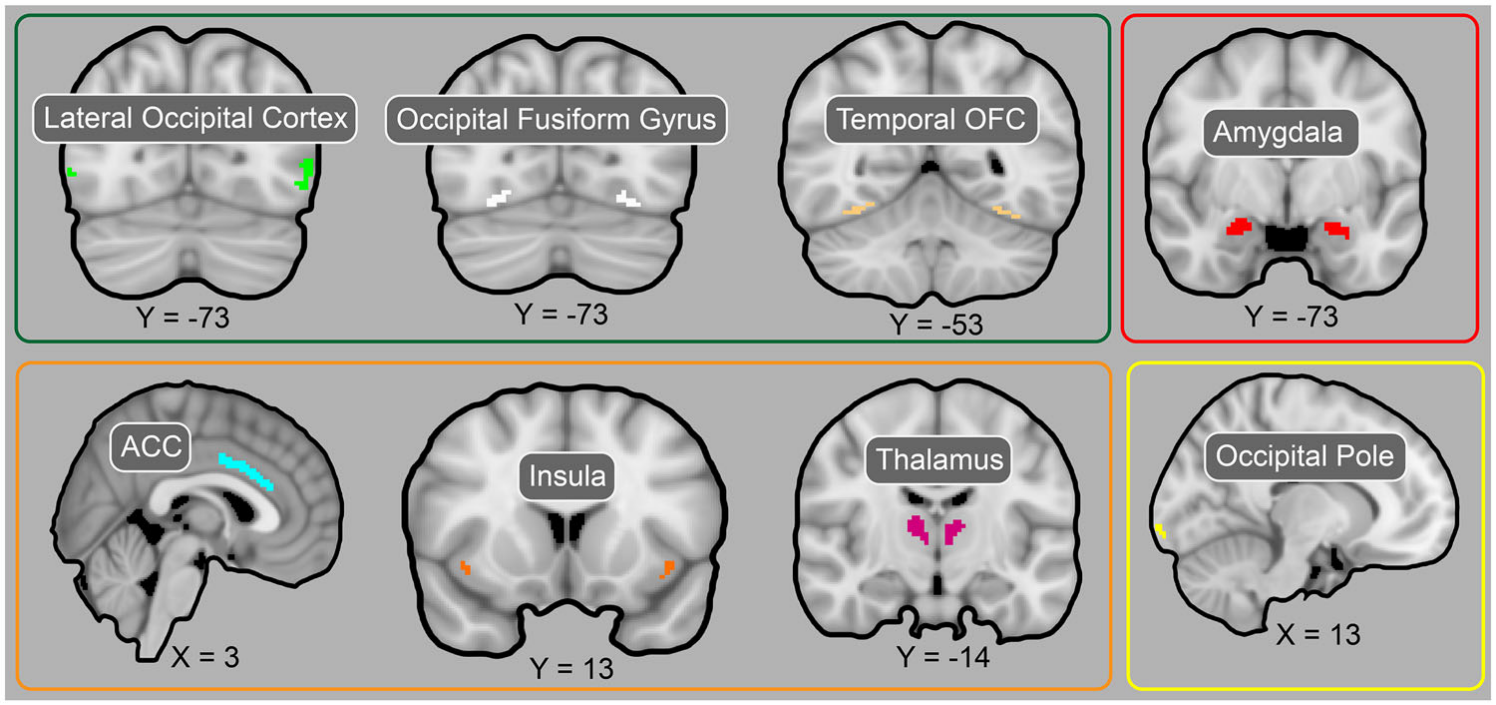
Harvard‐Oxford atlas regions of interest (ROIs) (thresholded) used in the functional magnetic resonance imaging (fMRI) study. Regions in the green box represent visual evidence accumulators, red represents amygdala, orange represents moment‐of‐decision, and yellow represents visual sensory. OFC=occipital fusiform cortex; ACC=anterior cingulate cortex.

Regions of interest (ROIs) were selected a priori, primarily based on previous findings of regions that showed evidence accumulation and MoD/recognition profiles in a gradual reveal paradigm (Ploran et al., 2007). These ROIs were chosen from the FSL‐distributed Harvard‐Oxford (HO) atlas (Figure 3), such as the amygdala and by selecting ROIs whose labels in the HO atlas matched the Ploran regions described as sensory processors, accumulators, and moment‐of‐recognitions. Although the HO atlas separates each ROI by hemisphere, we did not anticipate differences across hemispheres and therefore combined them. Given that the HO atlas is probabilistic, meaning that each voxel is given an ROI membership likelihood value, we thresholded each ROI to ensure that the surviving voxels (∼100 across both hemispheres) had a strong membership likelihood value to the specific ROI. Thresholding varied from 65% to 90%, as high thresholding (i.e., 90%) for certain ROIs would result in no voxels surviving, and low thresholding (i.e., 65%) for other ROIs would result in an extremely large number of voxels. Information regarding the selected ROIs can be found in Table 1.

**TABLE 1:** Region of interest (ROI) coordinates. ROI details are provided for each hemisphere; however, hemispheres were combined for analyses. The extent of each ROI was determined based on a % threshold value. The ROI size is based on 2 × 2 × 2 mm voxels.

(1) Center of mass coordinates for ROIs corresponding to evidence accumulator neural profiles						
Anatomical ROI	L/R	*x*	*y*	*z*	% Threshold	ROI size
Lateral occipital cortex (inf division)	L	−50	−73	−2	83	64
Lateral occipital cortex (inf division)	R	−52	−70	−2	83	31
Occipital fusiform gyrus	L	−27	−76	−14	65	42
Occipital fusiform gyrus	R	28	−73	−14	65	54
Temporal occipital fusiform cortex	L	−31	−52	−18	72	25
Temporal occipital fusiform cortex	R	33	−49	−18	72	74
Left = left, R = right. Coordinates are in MNI space						
(2) Center of mass coordinates for ROIs corresponding to moment‐of‐decision neural profiles						
Anatomical ROI	L/R	*x*	*y*	*z*	% Threshold	ROI size
Cingulate gyrus (anterior division)	L	−1	17	25	91	41
Cingulate gyrus (anterior division)	R	3	−20	24	91	70
Insula cortex	L	−39	2	−4	78	46
Insula cortex	R	40	4	−4	78	45
Thalamus	L	−9	−18	7	100	166
Thalamus	R	10	−17	8	100	142
Left = left, R = right. Coordinates are in MNI space						
(3) Center of mass coordinates for amygdala ROI neural profiles						
Anatomical ROI	L/R	*x*	*y*	*z*	% Threshold	ROI size
Amygdala	L	−23	−5	−18	90	45
Amygdala	R	−24	−3	−18	90	64
Left = left, R = right. Coordinates are in MNI space						
(4) Center of mass coordinates for ROIs corresponding to visual sensory neural profiles						
Anatomical ROI	L/R	*x*	*y*	*z*	% Threshold	ROI size
Occipital pole	L	−12	−100	−1	72	45
Occipital pole	R	16	−99	3	72	59
Left = left, R = right. Coordinates are in MNI space						

Following fMRIPrep pre‐processing and ROI generation, Nilearn's *NiftiMasker()* function was used to extract the ICA‐AROMA denoised time series residuals (automatic 6‐mm FWHM spatial smoothing applied) from each generated ROI mask. The extracted residuals were standardized (*z*‐scored) and detrended. Additionally, confounds provided by fMRIPrep were included as a form of nuisance regression. These confounds included the global signal, cerebral spinal fluid, white matter, cosine, and non‐steady state volume outliers, including all associated derivative and quadratic terms. Motion confounds were not included because we extracted the ICA‐AROMA denoised time series, which have already had motion confounds applied (Pruim et al., 2015) and including motion confounds again may reintroduce noise. All confounds were standardized by Nilearn and fit to the data.

A linear interpolation was then applied on a trial‐by‐trial basis to conform the residuals from each trial to a common length based on the mean decision response time of each salience condition. Importantly, interpolation was applied to residuals from trial onset to the decision response (pre‐decision interval) because that was the part of the residuals that varied in length from trial to trial; no interpolation was applied to the 10‐s post‐decision response period as it was unaffected by response time. This included shifting the residuals by 5 s to account for the (3–6 s) BOLD hemodynamic response delay. Additionally, the interpolated residuals for each trial were zero‐locked such that the parameter estimate value began at zero.

Lastly, a finite impulse response (FIR) function was applied to the interpolated residuals using the linalg.lstsq() function from Python's Numpy package. FIR was employed to summarize the profiles of the extracted residuals across subjects. Given the emphasis on accuracy over speed and the straightforward nature of the task, only correct decision trials were included for analyses (average subject accuracy >90%).

It should be noted that analyzing the drift rate and decision threshold involved measuring the salience conditions residuals, from the minimum parameter estimate within the first five trial seconds to the highest magnitude (i.e., maximum parameter estimate) within 3 plus/minus seconds of each salience condition average decision time. The determination of the 3 plus/minus seconds window was data driven, that is, a local maximum could be found within a window of that width for all subjects.

## 3 RESULTS

### 3.1 Behavioral

**FIGURE 4:**
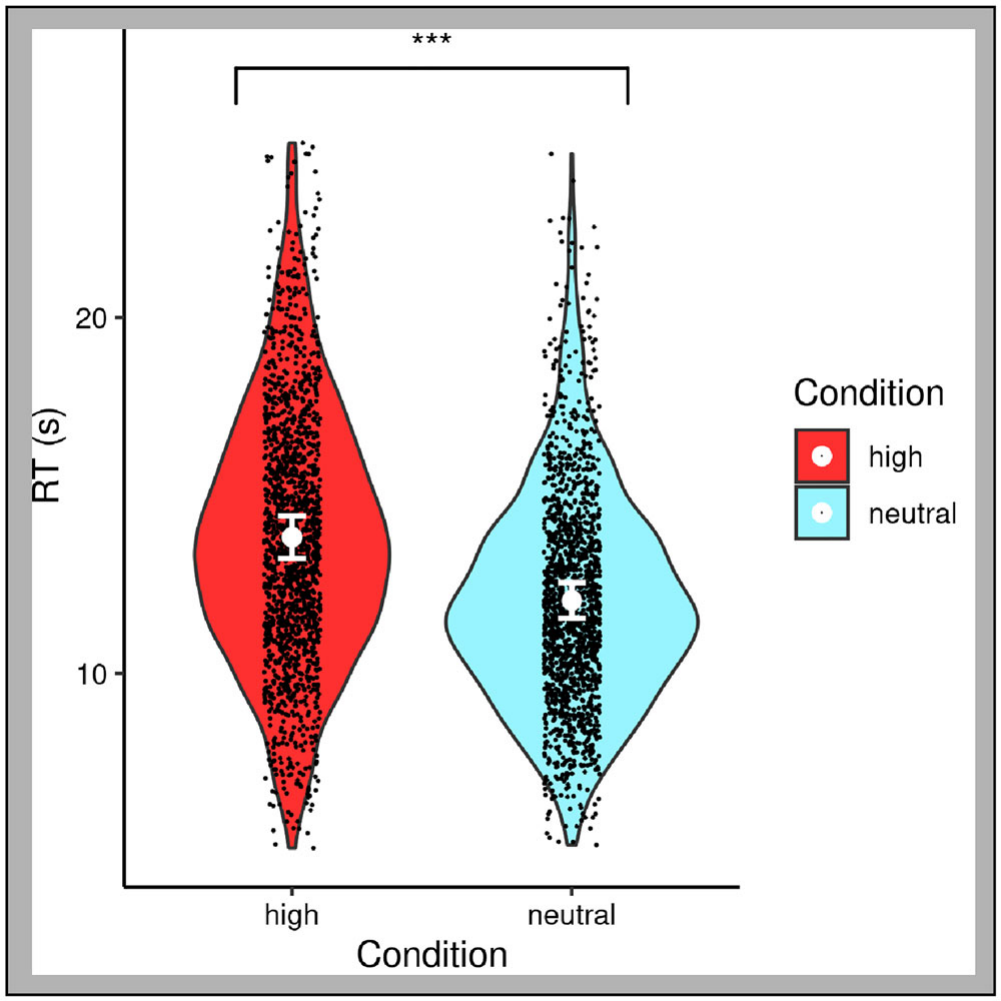
Violin plots of decision time as a function of affective salience condition. Error bars are in standard errors. Black dots represent values for each trial of each subject, and the larger white dots represent the mean value for each affective salience condition. ***p < .001. RT=reaction time.

A paired‐samples *t*‐test was performed to compare decision response times to affective salience conditions (Figure 4), and a significant difference was found; *t*(35) = 15.06, *p* < .001. As expected, based on the assumption that high salience would produce a distractor effect, response times for the high salience (mean = 13.83 s, SD = 3.58) body parts were slower than for the neutral body parts (mean = 12.05 s, SD = 3.01).

### 3.2 Drift rate (fMRI)

**FIGURE 5:**
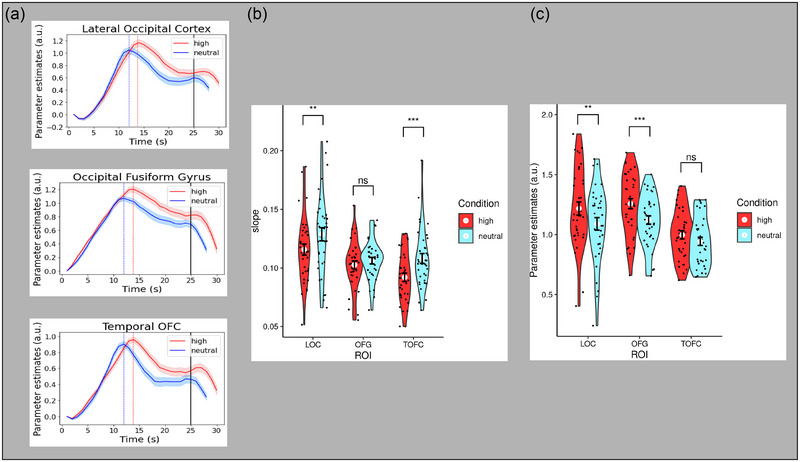
(a) Time series profiles for visual accumulator regions of interest (ROIs) as a function of ROI and salience condition. Blue vertical line = mean decision reaction time (RT) for neutral salience condition. Red vertical line = mean decision RT for high salience condition. Black vertical line = gradual reveal end. (b) Violin plots of slope (drift rate) as a function of ROI and salience condition for the visual accumulator ROIs. (c) Violin plots of peak magnitude (decision threshold) as a function of ROI and salience condition for the visual accumulator ROIs. a.u. = arbitrary units. Black dots represent values for each subject, and white dots represent the mean value for each affective salience condition. Error bars are in standard errors. **p < .01; ***p < .001. ns, nonsignificant.

To assess drift rate, we calculated the slopes of the salience conditions from the minimum parameter estimate within the first 5 trial seconds to the highest magnitude (i.e., maximum parameter estimate) within 3 plus/minus seconds of each salience condition average decision time. To test the hypothesis that affective salience affected the drift rate (slope) of evidence accumulation, a 3(ROI: accumulator ROIs) × 2(salience condition: high, neutral) analysis of variance (ANOVA) with subjects modeled as a random factor was performed. The three MoD ROIs were excluded from this analysis because the drift rate is a parameter that only effectively describes the evidence accumulator profile. There was a significant main effect of affective salience (*F*_(1, 175)_ = 15.59, *p =* .0001), with steeper slopes in the neutral compared to the high salience condition (Figure 5b). No significant interaction between affective salience and accumulator ROI was observed (*F*_(2, 175)_ = 1.82, *p =* .17). Thus, the distracting effect of task‐irrelevant high affective salience slowed the drift rate in all visual accumulator ROIs, with the exception of the occipital fusiform gyrus (OFG).

### 3.3 Decision threshold (fMRI)

**FIGURE 6:**
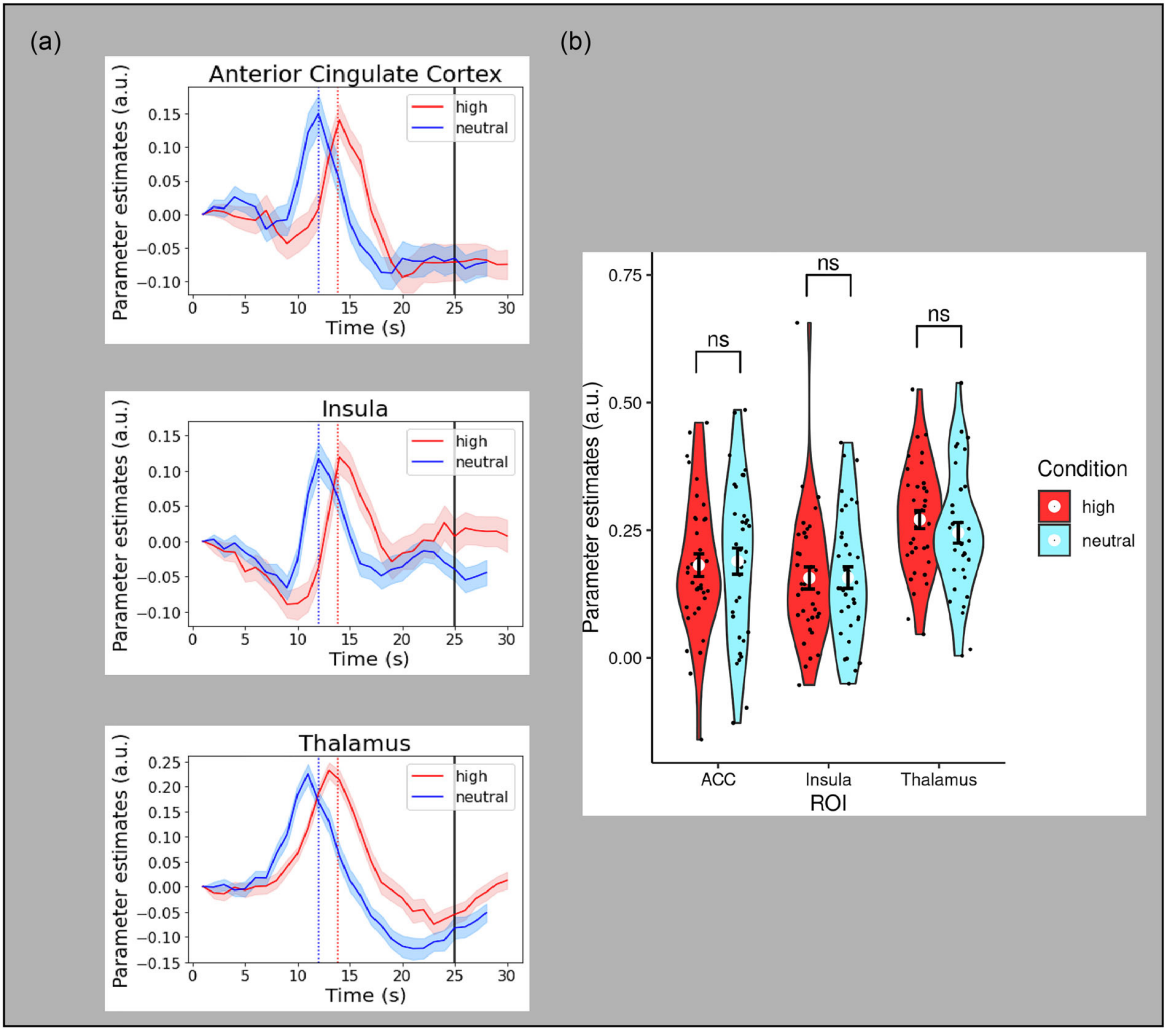
(a) Time series profiles for the moment‐of‐decision regions of interest (ROIs) as a function of ROI and salience condition. Blue vertical line = mean decision reaction time (RT) for neutral salience condition. Red vertical line = mean decision RT for high salience condition. Black vertical line = gradual reveal end. (b) Violin plots of peak magnitude (decision threshold) as a function of ROI and salience condition for the moment‐of‐decision ROIs. Black dots represent values for each subject, and white dots represent the mean value for each affective salience condition. Error bars are in standard errors. a.u., arbitrary units; ns, nonsignificant.

To assess decision thresholds (peak magnitude), we calculated peak parameter estimates within 3 plus/minus seconds of the salience condition decision time in each trial's interpolated time series. Trials were excluded if the maximum parameter estimate was less than zero, or if it occurred within the first 5 seconds of the gradual reveal. To test the hypothesis that affective salience modulated decision thresholds, we conducted two separate ANOVAs with subjects modeled as a random factor: a 2 (salience condition: high, neutral) × 3 (accumulator ROI: lateral occipital cortex (LOC), OFG, and temporal occipital fusiform cortex (TOFC)) and a 2 (salience condition: high, neutral) × 3 (MoD ROI: anterior cingulate cortex (ACC), insula, thalamus). These separate ANOVAs were conducted because we did not anticipate similar decision threshold findings between these distinct functional groups of ROIs (accumulators, MoD). There was a significant main effect of affective salience condition on decision threshold magnitude for the accumulator ROIs (*F*_(1, 175)_ = 11.60, *p =* .0008) (Figure 5c), but not for the MoD ROIs (*F*_(1, 175)_ = 0.13, *p =* .72) (Figure 6b). Given the significant main effect for the accumulator ROIs, we further examined these ROIs by performing three pairwise comparisons with Bonferroni correction to assess differences across salience conditions for the three regions of interest: LOC, OFG, and TOFC. Two of the three accumulator ROIs showed significantly greater peak magnitude for the high salience condition compared to the neutral condition: LOC (*t*(35) = 3.48, *p =* .004) and OFG (*t*(35) = 5.45, *p* < .0001). There was no significant difference in the other accumulator ROI, TOFC (*t*(35) = 2.49, *p =* .053). Thus, high affective salience induced a higher decision threshold in two of the three visual accumulator ROIs but in none of the MoD ROIs.

### 3.4 Amygdala profile (fMRI)

**FIGURE 7:**
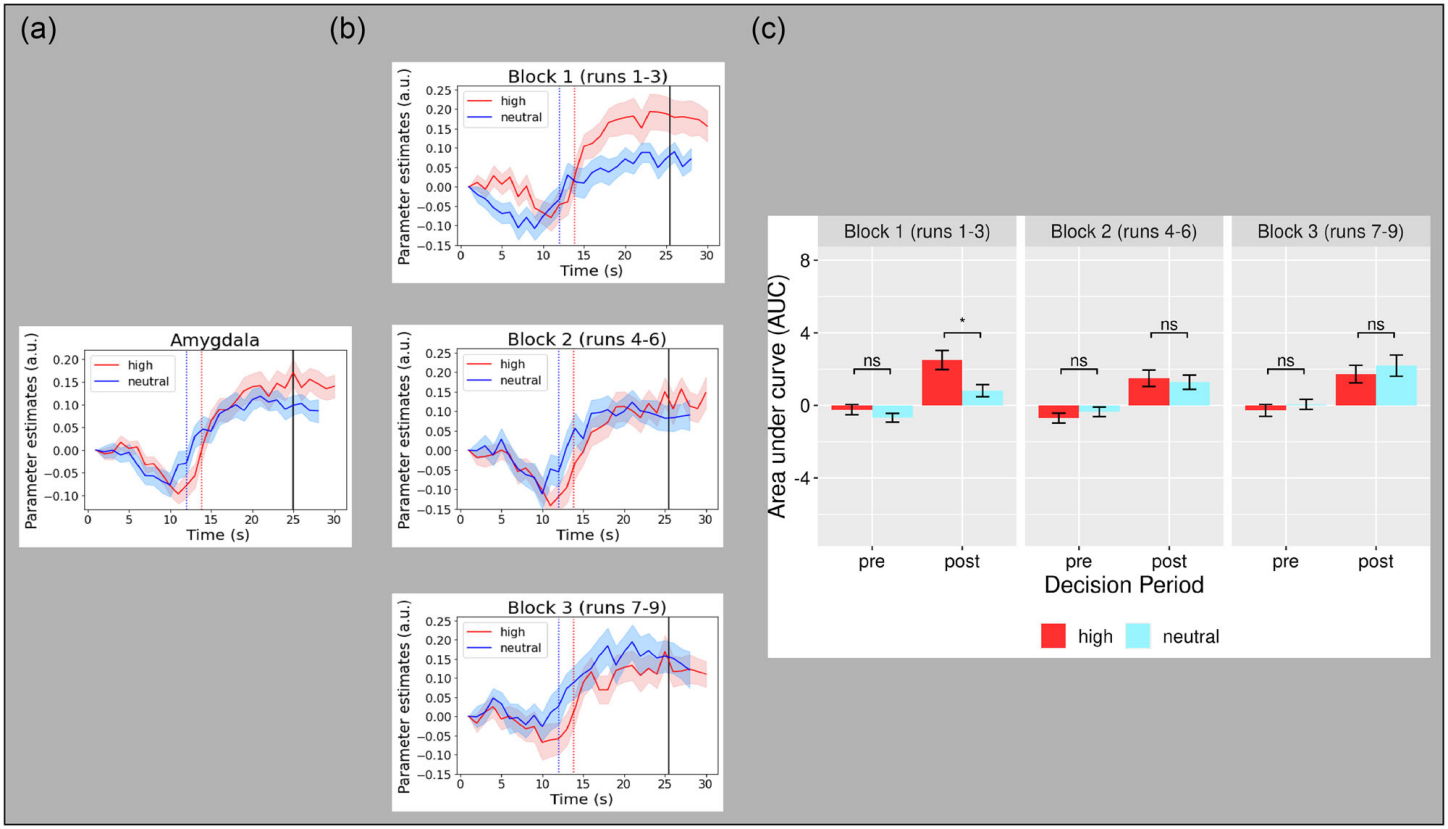
(a) Time series profile for the amygdala region of interest (ROI) as a function of salience condition. Blue vertical line = mean decision RT for neutral salience condition. Red vertical line = mean decision reaction time (RT) for high salience condition. Black vertical line = gradual reveal end. (b) Time series profiles for the amygdala ROI as a function scan block and salience condition. Blue vertical line = mean decision RT for neutral salience condition. Red vertical line = mean decision RT for high salience condition. Black vertical line = gradual reveal end. (c) Area under the curve (AUC) as function of scan block, decision period, and salience condition for the amygdala ROI. Error bars are in standard errors. *p < .05. ns, nonsignificant, a.u., arbitrary units.

It was important to assess the temporal profile of activation in the amygdala because of the consistent link between it and stimuli with high affective salience (Adolphs et al., 1995; Davis, 1992; Davis & Whalen, 2001; LeDoux, 2003). However, because previous studies that used gradual revealing had not assessed the amygdala, our approach was more exploratory than for the evidence accumulator and MoD ROIs. Thus, rather than using peak parameter estimate magnitudes and slope measures, we used a more “shape‐agnostic” approach by measuring the pre‐ and post‐decision area under the curve (AUC). Pre‐decision AUC started at trial onset and ended at the average decision time of each salience condition time series. Post‐decision AUC started at the decision time of each salience condition and ended at the conclusion of the time series. As Figure 7a demonstrates, the temporal profile in the amygdala did not subjectively match either the evidence accumulator or the MoD profiles seen in the other ROIs, nor did it match any of the other profile types documented previously, such as late positives, negatives, or bi‐modals (Ploran et al., 2007).

To more objectively evaluate the profile, pre‐ and post‐decision AUC values were modeled with a 2 (salience condition: high, neutral) × 2 (decision period: pre‐, post‐decision) ANOVA with subjects modeled as a random factor. The model produced a significant main effect of decision period (*F*_(1, 382)_ = 95.97, *p* < .0001), with post‐decision AUC greater than pre‐decision AUC, but no significant main effect of affective salience condition (*F*_(1, 378)_ = 0.96, *p =* .33) or interaction between salience condition and decision period (*F*_(1, 378)_ = 1.81, *p =* .18). The lack of a significant main effect of affective salience was unexpected because the amygdala is known to reliably produce greater activation with aversive stimuli compared to neutral. However, it is also well documented that strong habituation effects occur in the amygdala after repeated exposure(s) of affective salient stimuli, such that amygdala activation is attenuated over time (Breiter et al., 1996; Fischer et al., 2000; Wright et al., 2001). Therefore, it is possible that combining early trials where salience had a significant effect with later trials after the salience effect had habituated may have concealed the effects of salience. It is even possible that the gradual reveal paradigm may have heightened the influence of habituation.

Although admittedly investigatory, we decided that analyzing the effect of habituation on the salience effect in the amygdala was warranted. The decision to pursue a further analysis was also motivated by the results of a separate behavioral validation (see Supporting Information), which demonstrated that the specific high affective salience stimuli used in the experiment successfully elicited an aversive behavioral response from subjects. To analyze the influence of habituation on salience effects in the amygdala, time series were divided by their runs into separate scan blocks, each consisting of three runs. AUC measures for pre‐ and post‐decision were calculated separately for each scan block. The AUC measurements were submitted to a 3 (scan block: runs 1–3, runs 4–6, runs 7–9) × 2 (salience condition: high, neutral) × 2 (decision period: pre, post), repeated measures ANOVA, with subjects as a random factor. Significant main effects of affective salience (F_(1, 378)_ = 9.48, *p =* .002) and decision period (F_(1, 378)_ = 14.62, *p =* .0002) were found, along with a significant interaction between scan block and affective salience (*F*_(1, 378)_ = 8.54, *p =* .004). Given the notable temporal profile differences between the pre‐ and post‐decision period (Figure 7a), we next conducted six pairwise tests, with Bonferroni correction, to assess differences between affective salience conditions, one for each of the three scan blocks in each of the pre‐ and post‐decision intervals. The high affective salience condition produced significantly more activation than neutral in the post‐decision interval of the first scan block, *t*(34) = 2.92, *p =* .037 (Figure 7b). The other five tests were nonsignificant. Although investigatory, this analysis suggests that the amygdala response here may in fact be consistent with previous literature, but that habituation effects masked the effect salience condition. It also suggests that habituation effects with high affective salience may be more pronounced when using a gradual reveal paradigm. These findings should be considered in future studies using gradual revealing methods to assess amygdala responses.

In addition to these neural findings related to potential affective habituation, we also assessed whether differential reaction time effects for the high salience condition attenuated across the scan blocks. Reaction times, standard deviations, and errors across the three scan blocks are as follows: 13.91, 13.87, and 13.66 s for reaction time; 3.55, 3.73, and 3.43 for standard deviations; and 0.15, 0.16, and 0.16 for standard errors. Analysis with a one‐way ANOVA did not produce significant results for the reaction times, indicating no significant reaction time changes in the high salience condition across scan blocks.

### 3.5 Sensory profile (fMRI)

**FIGURE 8:**
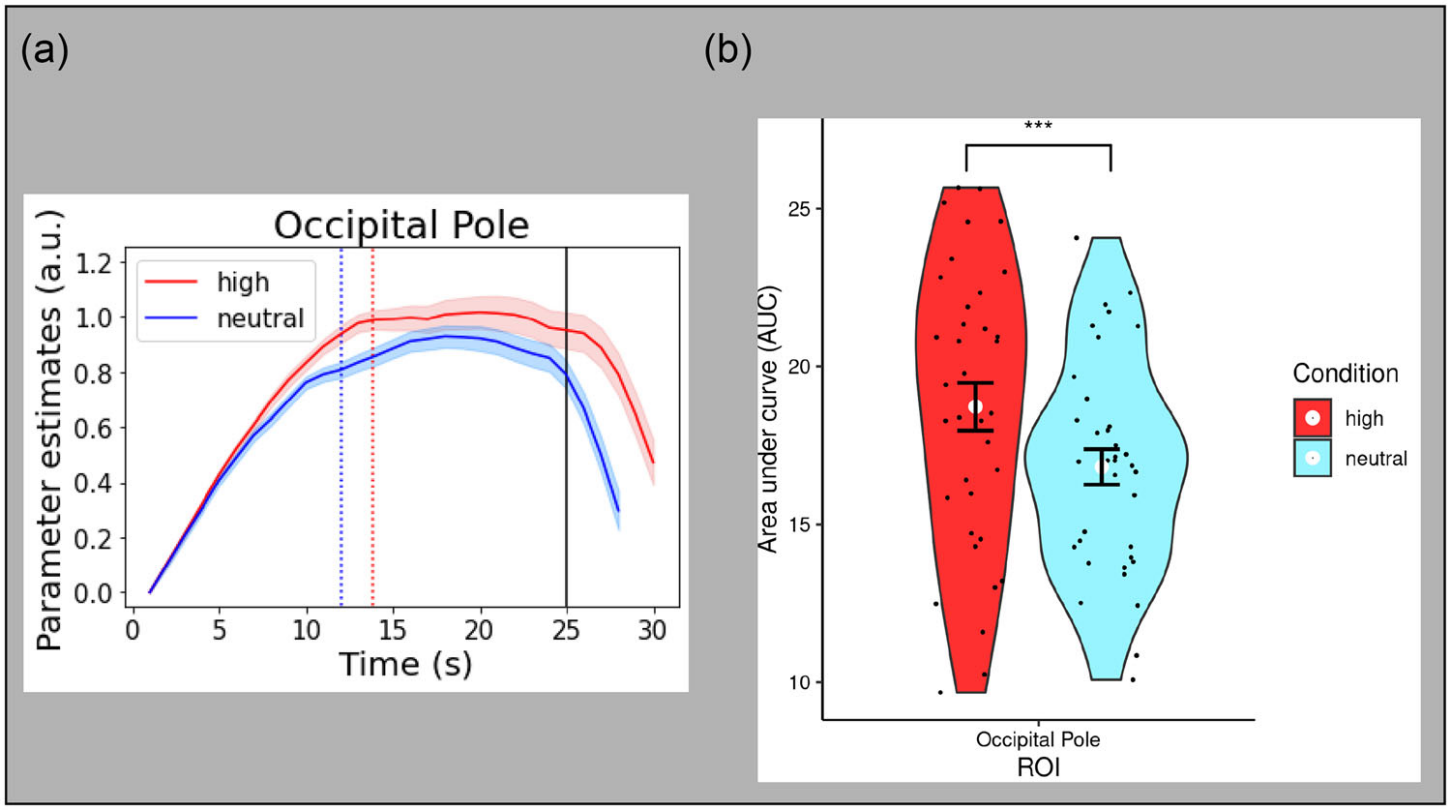
(a) Time series profile for the sensory region of interest (ROI) as a function of salience condition. Blue vertical line = mean decision reaction time (RT) for neutral salience condition. Red vertical line = mean decision RT for high salience condition. Black vertical line = gradual reveal end. (b) Violin plot of area under the curve (AUC) as a function of salience condition for the sensory ROI. Black dots represent values for each subject, and white dots represent the mean value for each affective salience condition. Error bars are in standard errors. a.u., arbitrary units.

Unlike evidence accumulator and MoD ROIs, the temporal profiles of sensory regions continually increase throughout the entire gradual revealing process and do not have features that are related to the decision time (Ploran et al., 2007). Thus, our analysis of the occipital pole ROI used a similar shape‐agnostic approach as the amygdala by measuring the AUC from stimulus onset until the end of the gradual reveal. An ANOVA with subjects modeled as a random factor was performed, producing a significant main effect of salience condition (*F*_(1, 35)_ = 19.45, *p* < .0001), with high salience time series AUC greater than neutral AUC (Figure 8). This finding suggests a sensitivity to the manipulation of object characteristics (via affective salience) within early sensory regions.

## 4 DISCUSSION

This study examined the influence of affective salience on object categorization decisions by analyzing the temporal activation profiles of multiple brain regions and, in particular, analyzing the parameters (drift rate, decision threshold) of evidence accumulation profiles in the visual cortex. Behaviorally, we found that the addition of task‐irrelevant aversive visual features to stimuli induced a distractor effect, demonstrated by longer decision times for high affective salience. Imaging results showed that affective salience influenced both drift rate and decision threshold in evidence accumulator ROIs, with high salience attenuating drift rate (reducing the quality of evidence) while increasing the decision threshold (or stopping rule). Rather than arbitrate between the hypotheses shown in Figure 1, these findings suggest a combination of both hypothesized mechanisms. This is the first demonstration, to our knowledge, of an experimental manipulation of sensory data and also the first demonstration, to our knowledge, of changes to the neural decision threshold in a gradual reveal paradigm. These two findings advance our knowledge of the underlying neural mechanisms of object categorization decisions.

### 4.1 Differences in quality of evidence reflected by drift rate

The primary purpose of this study was to analyze the influence of affective salience on the parameters of evidence accumulation in the visual cortex. Previous studies using gradual reveal paradigms have characterized this “accumulation” profile as activation that increases from trial onset until the MoD, which is also the time when activation reaches peak magnitude, and then decreases from the MoD to the end of the trial, even though sensory evidence continues to build after the decision is made (James et al., 2000; Ploran et al., 2007; Wheeler et al., 2008). The steadily increasing activation during the pre‐decision period can be characterized by its slope or drift rate. In the previous studies, stimuli that produced quicker decisions (i.e., shorter decision time) also produced steeper slopes. Because the magnitude of peak activation was always the same regardless of decision time (see discussion on decision threshold in Section 4.2), steeper slopes always led to earlier time of peak activation. Relating these findings to drift‐diffusion models suggests that the quicker decisions were due to a better quality of evidence contained in the sensory data or the memory representations. The study by James et al. demonstrated this change in slope with a design that manipulated memory representations (with a priming paradigm) while holding physical stimulus characteristics constant. Together, previous studies suggested that differences in neural drift rate were best explained by the improved quality of evidence in memory representations.

Consistent with this previous work, the results of our manipulation of affective salience (Figure 5b) also showed differences in neural drift rate (albeit in combination with differences in decision threshold; see below). Body parts with task‐irrelevant features of mutilation (high affective salience) were categorized more slowly (a distractor effect), showed a shallower drift rate (slope), and showed a longer time to peak magnitude. Relating these results to drift‐diffusion models suggests that adding task‐irrelevant features to the stimuli to increase affective salience decreased the quality of evidence that was extracted from the task‐relevant sensory data.

### 4.2 Differences in stopping rule reflected by decision thresholds

With drift‐diffusion models, the variability of decision thresholds/boundaries is largely dependent on context. Speed‐accuracy tradeoffs are typically used as an example, where emphasis on speed induces a lower decision threshold, and an emphasis on accuracy leads to a higher decision threshold (Bogacz et al., 2006; Ratcliff et al., 2016). In our experiment, we purposefully emphasized accuracy over speed, instructing participants to make their decision response once they felt “confident” in their ability to accurately categorize the stimuli; therefore, it could be expected that the decision thresholds across salience conditions would be equivalent. However, an alternative hypothesis (Figure 1b) was that the decision threshold would be raised by high affective salience, which would explain the well‐known finding of increased BOLD activation with high salience stimuli in visual cortex.

Our results (Figure 5c) demonstrated that high affective salience did produce greater peak activation at the MoD (i.e., the neural decision threshold), even though this was paired with a shallower drift rate. Relating this finding to drift‐diffusion models, it is suggested that task‐irrelevant, high affective salience may artificially shift the stopping rule to be more conservative (i.e., more evidence is required for a decision). Of course, in this experiment, the visual features that increased salience were both high salience and task‐irrelevant. Therefore, it is unclear if the effect on decision threshold would also occur with any task‐irrelevant features/information or if the task‐irrelevant data need to be high in salience. One possibility is that the presence of task‐irrelevant features (i.e., noise/uncertainty) in the sensory data that are deemed high in “significance” (Adolphs, 2010) may cause the visual system to use a more conservative threshold for categorization decisions. This possibility is even more plausible given the experiment instructions, which deemphasized speed.

An interesting finding of ours was that statistically significant decision threshold differences were observed in the accumulator ROIs but not the MoD ROIs. Although MoD regions are functionally classified by their sensitivity to decision/response behaviors, evidence accumulator regions naturally code decision threshold parameters in addition to drift rate (Ploran et al., 2007). While our study was designed to examine the influence of negative affect as a distractor on visual accumulator neural profiles, it will be necessary for future studies to address the degree to which the decision threshold differences are driven by visual processes, or “downstream” processes that presumably occur in non‐visual regions across the cortex.

### 4.3 Temporal profile in the amygdala

Although the primary goal of this study was to assess the influence of affective salience on evidence accumulation in visual cortex, the consistent association between affective salience and the amygdala in previous work led us to examine its temporal profile (Pessoa et al., 2005). In fact, to our knowledge, the current study is the first to report on the temporal profile of the amygdala during gradual revealing. Due to the lack of previous data, we did not have an a priori hypothesis about how the amygdala would respond to affective salience differentially before or after a decision, nor even a hypothesis about the shape of its temporal activation profile. As such, the amygdala time series were subjected to a more “shape‐agnostic” exploratory analysis than that of the accumulator and MoD ROIs. This analysis revealed that the amygdala time series showed little similarity to any of the temporal profiles characterized in previous work (James et al., 2000; Ploran et al., 2007). The amygdala temporal profile resembled a step function, with a step‐up occurring at the MoD. Pre‐decision activation was close to zero, with a slight negative drift as it approached the MoD. Post‐decision activation was much higher than pre‐decision activation and appeared to asymptote as opposed to declining. This profile suggests that the amygdala is most sensitive to the decision point of the gradual reveal trial, which further suggests a high sensitivity to understanding objects in the environment (Palmeri & Gauthier, 2004).

An unexpected finding in the amygdala, however, was the lack of significant differences between affective and neutral salience conditions (Figure 8a). Our first consideration was the possibility that our specific stimulus set did not invoke the appropriate affective responses in subjects. The data from a separate behavioral validation (see Supporting Information), where subjects rated the subjective arousal (salience) and valence (pleasant vs. unpleasant) of the same stimuli used in this experiment, effectively ruled out this possibility. A second consideration was based on evidence that amygdala activation with affective salient stimuli is attenuated when attentional demands are high (Palmeri & Gauthier, 2004; Pessoa et al., 2005). Attentional demands are certainly different in a gradual reveal paradigm than when whole stimuli are displayed quickly; thus, attentional demand should be considered a plausible reason for the lack of salience effect in the amygdala. However, further testing would be required to determine more precisely if and how attentional demand was influencing the processing of affective salience.

A third possible consideration was based on the idea that the role of the amygdala has too oftentimes been associated solely with fear detection; it has also been characterized as a “relevance detector” (Adolphs, 2010; Sander et al., 2003). This may be applicable in the case of the current experimental paradigm because the features used to produce the high affective salience were task‐irrelevant—the task was to categorize the body parts and the mutilation features were not useful for performing the task. If the amygdala is detecting “relevance” and the features that produced high salience were not relevant to the task, that could explain the lack of difference in amygdala activation between salience conditions. A fourth and final consideration is the well‐known habituation of affectively salient images after repeated exposure (Breiter et al., 1996; Fischer et al., 2000; Wright et al., 2001), which may even have been more pronounced with the gradual reveal paradigm. This possibility was confirmed to a degree. When the amygdala time series were binned into scan blocks (three consecutive runs out of nine total), the typical hyperactivation to salient stimuli was found during the post‐decision period in the first scan block (Figure 7c) but disappeared in the second and third scan blocks. Although based on a post hoc analysis, the results showed that habituation occurred, making habituation a strong candidate as the explanation for the lack of difference between salience conditions in amygdala activation in this experiment. However, whether the lack of difference was due to habituation, task relevance, attentional demand, or some combination of the three, more research is clearly needed to fully understand the amygdala temporal profile and its sensitivity to affective salience.

An interesting aspect of the amygdala temporal profile was that the greater activation with aversive stimuli in the first scan block was seen only after the MoD. This result is pertinent to an ongoing debate in the emotion literature regarding whether or not emotional processing occurs in the absence of conscious awareness (for review, see Pessoa & Adolphs, 2010). While our study did not seek to directly inform this debate, the results suggest that under the specific conditions of this study, pre‐awareness does seem to be a requirement for differences in salience to be detected in the amygdala. Even though this analysis was performed post hoc, it is interesting to speculate that the lack of a salience effect in amygdala activation before the MoD (i.e., pre‐awareness) in the first scan block is comparable to previous findings showing that the amygdala response to salient stimuli is constrained by awareness (Pessoa et al., 2002, 2005; Phillips et al., 2004).

The lack of pre‐decision differences between salience conditions in the amygdala is in contrast to the presence of pre‐decision differences between salience conditions in visual accumulator ROIs. These findings may inform a long‐standing debate about whether or not the amygdala has privileged access to “fear” stimuli via a sub‐cortical circuit that bypasses the usual hierarchical processing circuits in visual cortex (Pessoa & Adolphs, 2010). A consequence of this idea is that increased activation in visual cortex related to high affective salience (Bradley et al., 2003; Damaraju et al., 2009; Lang et al., 1998; Markovic et al., 2014) is thought to rely on feedback from the amygdala. In contrast, the current findings suggest that visual accumulators are sensitive to high affective salience before there is enough sensory data available to make a decision, whereas the amygdala appears to only differentiate high affective salience once a significant amount of sensory data is available.

An important note regarding the amygdala analyses was that AUC was calculated for analysis rather than slope (drift rate) or peak magnitude (decision threshold). The rationale for AUC was because it is a theory‐neutral measurement. With regard to drift‐diffusion models of evidence accumulation, measurements including slope (for drift rate) and peak magnitude (for decision threshold) correspond to the theoretical expectations of the evidence accumulator neural profiles (Ploran et al., 2007). However, the amygdala has not been studied in this context, nor has it been previously identified as an evidence accumulator. Indeed, the profiles of our amygdala results are quite dissimilar to the profiles exhibited in our evidence accumulators and other examined functional ROIs, reflecting instead a step‐like neural profile. Given these considerations, assessing the amygdala profile using slope or peak magnitude was deemed inappropriate in the context of evidence accumulation theories, and therefore AUC was instead utilized as it is a more theory‐agnostic approach.

### 4.4 Limitations

For this study, high affective salience was created by adding mutilation features to half of a set of body parts (hands and feet) stimuli. This manipulation was chosen because it was known to produce large changes in salience with relatively small visual featural changes and, importantly, without changes to the features that are fundamental to the body part categorization task. However, mutilations do change the visual characteristics of body part objects in systematic ways, even if the changes are small. It is possible that the longer response times with high salience images were caused by these small changes to visual characteristics, rather than the effect of aversive salience. We consider this study a first attempt at assessing the influence of affective salience on neural accumulators, and future studies will need to generalize the effects found here to other manipulations of affective salience that do not rely on changing visual characteristics. Furthermore, future studies employing this or a similar paradigm would be wise to collect arousal ratings (as done in the Supporting Information validation), as well as skin conductance measurements to provide physiological support for their findings.

Additionally, while affective information can contain either a positive (i.e., appetitive) or negative (i.e., aversive) valence, in this study, only negative valence stimuli (e.g., gore, mutilations) were employed. The reason for this was due to the concern that including positive valence would not induce similar levels of arousal compared to negatively valenced stimuli (Lang et al., 1997). Thus, we focused on negative images to maximize the size of any potential effects. We acknowledge that our findings may therefore be specific only to negative affect and not necessarily generalize to positive affect; future studies will be needed to clarify this generalizability question.

A criticism of the gradual reveal paradigm for assessing neural accumulation is that the gradual increase of activation from trial onset to decision time may be an epiphenomenon induced by the paradigm itself (i.e., by the gradual increase of available information over time). This is a viable concern with the sensory ROI (Figure 8), where the temporal profile does appear to mimic the time course of the paradigm. However, the presence of the other profiles suggests that the parameters of all the profiles are reflecting neural mechanisms that are only measurable when processing is slowed down.

Regarding the parameters themselves, in this study, we were primarily interested in the drift rate and decision threshold, despite the fact that starting point bias is a parameter also commonly assessed in evidence accumulator models. We did not consider bias in this study because the exact mechanism underlying differences in start point is contested in the modeling literature, whereas the mechanisms underlying drift rate and threshold have repeatedly been related to neural markers, including those of gradual reveal paradigms. We suggest that a targeted investigation of the neural mechanisms of the starting point is needed before the starting point should be used as a dependent measure of neural evidence accumulation to assess experimental manipulations like the one here.

Regarding our amygdala findings, these were generated from measuring the entire amygdala region, despite that it is not a monolithic functional entity, but rather a collection of nuclei implicated in different functional processes. In this study, we were principally interested in the temporal profiles of the assessed functional ROIs (amygdala included) and thus prioritized temporal over spatial resolution. Future studies prioritizing spatial resolution will be needed in order to examine the roles and profiles of amygdala nuclei.

Finally, although a post hoc analysis of the amygdala profile suggested that there were strong habituation effects, there was no other type of measurement to produce converging evidence of habituation. Evidence of habituation could have also been assessed by collecting psychophysiological measures such as skin conductance responses, pupil dilation, or heart rate and also with behavioral ratings of salience, simultaneous with the BOLD measurements.

## 5 CONCLUSIONS

The primary aim of this study was to assess the influence of affective salience on the neural mechanisms of evidence accumulation during categorization decisions using a gradual reveal paradigm. Task‐irrelevant, high affective salience lengthened response times (a distractor effect). In visual accumulator ROIs, high salience lessened the drift rate and increased the decision threshold, suggesting neural mechanisms involving the extraction of lower quality evidence from the sensory data combined with a less conservative stopping rule. The temporal profile in the amygdala was found to be different from the previously documented profile classifications. It resembled a step function with a dramatic step up at the decision time. Unexpectedly, the amygdala was only sensitive to high affective salience in the post‐decision period for trials early in the experiment, suggesting a strong habituation effect. These findings advance our knowledge of how affective salience influences the neural mechanisms involved in categorization decisions and raises compelling questions about the role of awareness in emotion processing and about the direction of information flow between the amygdala and visual cortex.

## AUTHOR CONTRIBUTIONS

**Daniel J. Levitas**: Conceptualization; methodology; software; formal analysis; investigation; data curation; writing—original draft; writing—review and editing; visualization; project administration. **Kess L. Folco**: Conceptualization; software; formal analysis; writing—review and editing. **Thomas W. James**: Conceptualization; methodology; formal analysis; writing—original draft; writing—review and editing; supervision.

## CONFLICT OF INTEREST STATEMENT

The authors declare no conflicts of interest.

## FUNDING INFORMATION

This research was supported in part by Lilly Endowment, Inc., through its support for the Indiana University Pervasive Technology Institute. This material is based upon work supported by the National Science Foundation under Grant No. CNS‐0521433. This work was supported in part by Shared University Research grants from IBM, Inc., to Indiana University.

### PEER REVIEW

The peer review history for this article is available at https://publons.com/publon/10.1002/brb3.3312.

## Supporting information


**Figure S1**. Behavioral validation results. Participants rated images on two dimensions: arousal and valence. Valence rating values are displayed on the x‐axis and arousal (i.e., salience) rating values on the y‐axis. The distinct clustering of the affective salient and neutral images suggests that images with aversive features (high affective salience condition) were uniformly perceived as more highly arousing and of greater negative valence (i.e., unpleasant) than the non‐aversive images (neutral salience condition).

## Data Availability

All data and code associated with this study will be made publicly available.
