# Supplementary material for: Impact of aversive affect on neural mechanisms of categorization decisions
Source: Brain Behav. 2023 Nov 15;13(12):e3312. doi: 10.1002/brb3.3312 (PMC10726818; doi:10.1002/brb3.3312)
Supplement: Supplementary file 1 — Figure S1. Behavioral validation results. Participants rated images on two dimensions: arousal and valence. Valence rating values are displayed on the x‐axis and arousal (i.e., salience) rating values on the y‐axis. The distinct clustering of the affective salient and neutral images suggests that images with aversive features (high affective salience condition) were uniformly perceived as more highly arousing and of greater negative valence (i.e., unpleasant) than the non‐aversive images (neutral salience condition). [file BRB3-13-e3312-s001.docx]

***Supplementary Materials***

**fMRI:**

A video demonstration of an example trial during the fMRI experiment can be found at this link: <https://drive.google.com/file/d/1eURVPtjQoE01aHd8DclcV3wYpU1tTdDo/view?usp=sharing>

**Behavioral validation study:**

**1 | Introduction**

A behavioral validation of our stimulus set was prompted by the lack of an effect of affective salience condition in the Amygdala time series findings from the fMRI experiment. We had purposefully created our own stimulus set that would evoke as strong subjective difference between aversive and neutral affective salience as curated stimulus datasets such as the International affective picture system (IAPS; [(Lang et al. )](https://paperpile.com/c/AH2nrW/VAB6)), but that would do so with only small visual feature changes between the aversive and neutral conditions. However, unlike the IAPS dataset, our stimulus had not been validated, and the lack of significance difference between salience in the fMRI data in the Amygdala forced us to consider the possibility that no subjective difference between conditions had been achieved.

**2 | Materials and Methods**

*Stimuli*

The stimuli were identical to the ones presented during the fMRI experiment. Stimuli consisted of 100 images (and an additional six for practice) with 50 images of feet and 50 images of hands. Half of the appendages contained highly affective salient features, such as lacerations and mutilations. All images were resized to 600 x 600 pixels, with gray-scaled backgrounds in order to keep the emphasis on the appendage.

*Participants*

29 adults aged 18-23 (9 males; average age = 20.21 years, SD = 2.54) with normal or corrected-to-normal vision were recruited to participate in the study, which was approved by the Institutional Review Board of Indiana University. Five participants were removed from analysis due to data loss. Thus, the remaining cohort was 24 participants aged 18-22 (6 males; average age=21.98 years, SD = 2.44 years). Participants were compensated with course credit. None of the participants had participated in the fMRI experiment.

*Experimental paradigm*

Participants were seated in front of a Dell computer, where they were instructed to rate each image on two dimensions: How *arousing* the image was, and how *pleasant or unpleasant* it was (the valence). The arousing dimension was on a continuous scale of 0 to 100, with 100 being “*extremely strong arousal*” and 0 being “*extremely weak arousal*”. The pleasant/unpleasant dimension was on a continuous scale of -50 to 50, with -50 being “extremely unpleasant”, 0 being “neutral”, and 50 being “extremely pleasant”. Participants viewed each image for three seconds, and then made their selections by sliding a computer mouse. Screen resolution was 1280 x 1024 pixels, and participants sat approximately 18 inches from the computer screen.

**3 | Results**

Two planned paired t-tests were conducted on the ratings, one for the arousal score between the salience conditions, and the other for the valence score between the salience conditions. A significant difference was found for arousal ratings, t(23)=4.33, p<0.001, with the mutilated condition (mean=51.73, SD=34.05) showing greater arousal than the neutral condition (mean=20.77, SD=18.53). A significant difference was also found between the valence ratings, t(23)=-12.03, p<0.001, with the mutilated condition (mean=-33.75, SD=16.77) showing higher valence than the neutral condition (mean=5.01, SD=16.81).

**4 | Discussion**

The significant differences between stimulus conditions suggest that our manipulation of mutilation features did successfully produce strong differences in affective salience between the two conditions that should have differentially activated the amygdala. Thus, it was likely that some characteristic of the gradual revealing method was what limited differences in activation in the amygdala in the current study. We discuss these possibilities in the main article.

**5 | Figures**

***
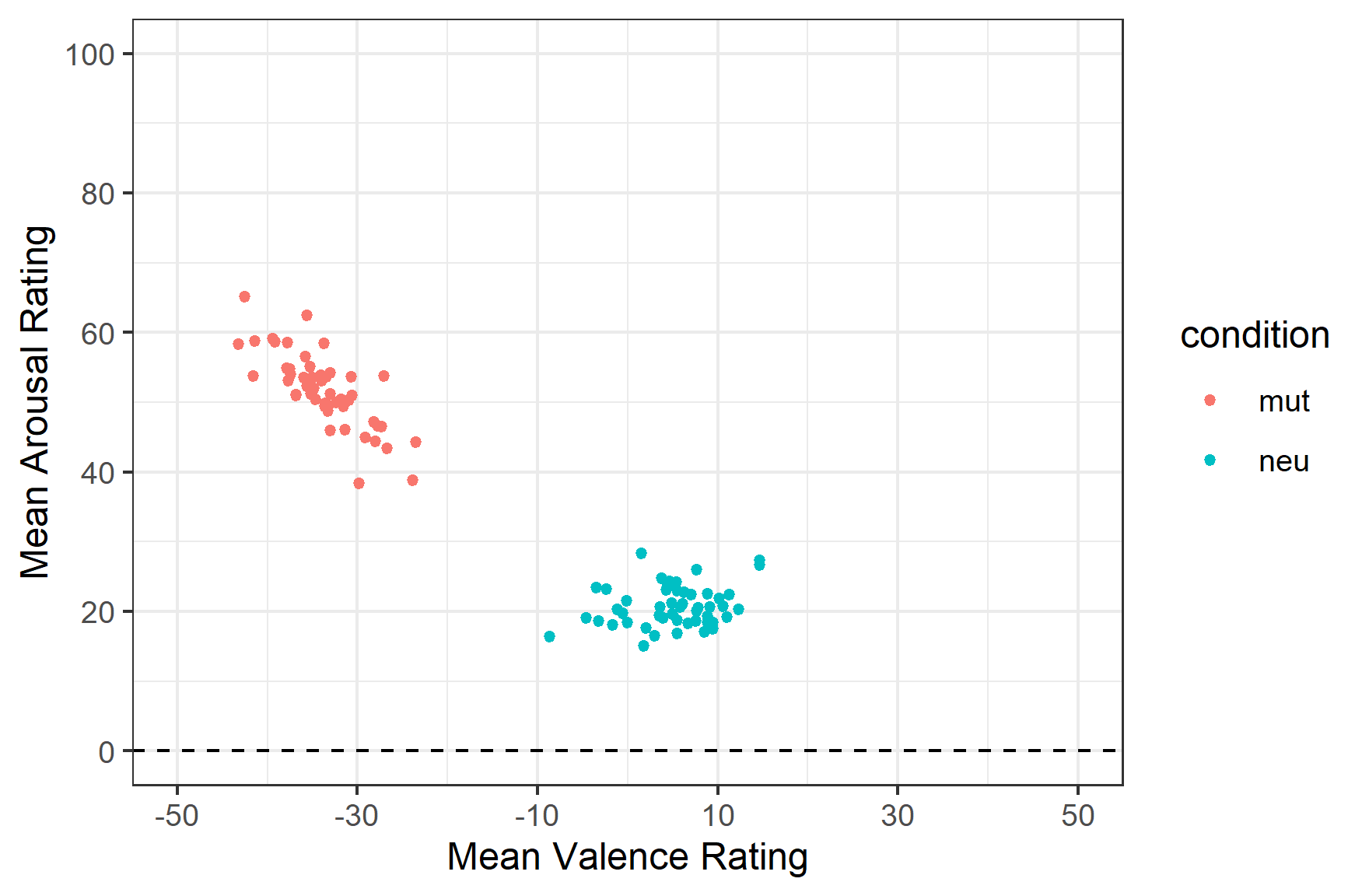
***

**Figure S1.** Behavioral validation results. Participants rated images on two dimensions: arousal and valence. Valence rating values are displayed on the x-axis and arousal (i.e. salience) rating values on the y-axis. The distinct clustering of the affective salient and neutral images suggests that images with aversive features (high affective salience condition) were uniformly perceived as more highly arousing and of greater negative valence (i.e. unpleasant) than the non-aversive images (neutral salience condition).
